# A free-living, walking-based, exercise programme, with exercise timed relative to breakfast, to improve metabolic health in people living with overweight and obesity: A feasibility study

**DOI:** 10.1371/journal.pone.0307582

**Published:** 2024-11-21

**Authors:** Jennifer S. Barrett, Anthony Crozier, Daniel J. Cuthbertson, Juliette A. Strauss, Anton J. M. Wagenmakers, Sam O. Shepherd

**Affiliations:** 1 Research Institute for Sport & Exercise Sciences, Liverpool John Moores University, Liverpool, United Kingdom; 2 Department of Cardiovascular and Metabolic Medicine, University of Liverpool, Liverpool, United Kingdom; 3 Metabolism & Nutrition Research Group, Liverpool University Hospitals NHS Foundation Trust, Liverpool, Merseyside, United Kingdom; Maastricht University Faculty of Health, Medicine and Life Sciences: Maastricht Universitair Medisch Centrum+, NETHERLANDS, KINGDOM OF THE

## Abstract

Optimising the timing of food intake relative to exercise may maximise the effectiveness of free-living exercise programmes on improvements in glycaemic control and cardio-metabolic health. This study aimed to assess the feasibility of a free-living, walking-based exercise programme and determine whether undertaking each exercise session *before* or *after* breakfast would most benefit longer-term metabolic health. Thirty-four people living with obesity (43±12 y, BMI 35.1±5.1 kg.m^-2^) undertook a 12-week walking-based programme, consisting of two continuous (30–60 min at 50% HR_max_) and two interval exercise sessions per week (30–60 min, alternating 3 min at 85% HR_max_ and 3 min at 50% HR_max_). Participants were allocated to exercise before (FASTED) or after (FED) breakfast (*n* = 17 per group). Feasibility (acceptability, adherence and compliance) to the exercise intervention were assessed, as well as changes in anthropometric variables, 24-hour continuous glucose monitoring, serum biochemistry including HbA1c, lipid profile and liver transaminases. Exercise adherence (FASTED: 93±4%, FED: 95±5%) and compliance (FASTED: 85±10%, FED: 88±10%) was high in both groups, and participants described exercise monitoring, programme structure and support as facilitators to this. Body mass, BMI, waist-to-hip ratio and HbA1c decreased similarly between groups (all *P*<0.01). However, serum ALT concentrations decreased after FASTED (-16± -14%; *P* = 0.001), but not FED training (-2 ± -4%; *P* = 0.720). We demonstrate that a free-living walking-based exercise programme, with exercise timed relative to breakfast can achieve high adherence and compliance and improve some anthropometric variables and HbA1c. Whether FASTED exercise can elicit greater improvements in liver health requires further investigation.

## Introduction

Regular exercise remains the cornerstone for people living with overweight or obesity to improve cardio-metabolic health and prevent the development of type 2 diabetes (T2D) [1–10] and there is consensus that exercise improves metabolic health in individuals with T2D [11, 12]. In this regard, current guidelines recommend undertaking ≥150 min of moderate-to-vigorous intensity aerobic exercise spread across 3–5 days per week [13, 14]. Supervised exercise programmes, employing moderate-to-vigorous intensity, aerobic exercise, improve the cardio-metabolic risk profile of people with overweight or obesity, including greater glycaemic regulation, increased insulin sensitivity, reductions in body and fat mass, better cardio-respiratory fitness, and a more favourable lipid profile [15–18]. Under free-living conditions, lower intensity, continuous-type exercise appears to be the preferred option for those individuals with low physical capabilities [19] when compared to high intensity, interval training and HIIT also has a lower adherence in free-living conditions [20]. However, studies using lower intensity exercise report little or no improvements in glycaemic control and insulin sensitivity [21–24], and walking-based exercise programmes rarely alter serum lipid profiles [25]. Moreover, there appears to be large differences in the response to low-intensity or walking-based exercise programmes aiming to improve insulin sensitivity and glycaemic control [26, 27]. Thus, strategies that can enhance the therapeutic benefits of walking-based exercise interventions warrant investigation.

Manipulating the timing of meal ingestion relative to exercise is an important consideration since this can impact the metabolic response to exercise and could enhance the long-term effectiveness of an exercise programme. For example, exercising before breakfast elicits high whole-body fat oxidation rates, stimulates hepatic glycogenolysis, and enhances adipose tissue lipolysis to increase fatty acid availability during exercise [28]. In contrast, exercising after breakfast (i.e., in the postprandial state) leads to lower glycaemic responses to subsequent meals compared to exercise undertaken before breakfast [29]. How these acute metabolic responses translate to changes in glycaemic control and insulin sensitivity following chronic training, with exercise undertaken before versus after breakfast is not yet clear. In healthy individuals, fasted exercise training for 6 weeks leads to greater improvements in insulin sensitivity compared to training after breakfast (i.e., in the fed state) [30, 31]. In individuals living with obesity, insulin sensitivity was increased following 6 weeks of fasted exercise training [32], whereas in individuals living with T2D, greater improvements in HbA1c following 12-weeks of training was observed where exercise was undertaken after breakfast [16]. Importantly, obesity is also associated with accumulation of hepatic fat +/- inflammation and fibrosis, [33], now known as metabolic dysfunction-associated steatotic liver disease (MASLD), while intermittent fasting, with or without exercise, has been shown to improve liver biochemsitry [34]. Importantly, all chronic training studies comparing the effect of exercising in the fed or fasted state on cardio-metabolic health outcomes have been undertaken in a laboratory setting, with strict timing of exercise relative to food intake. These studies are not directly translatable to everyday living and so whether the timing of exercise relative to the first meal of the day determines the response to an exercise training programme under free-living conditions is not known.

Manipulating the timing of food intake, on a population basis, maybe feasible given that this approach could improve health parameters, considering the widespread adoption of related dietary interventions such as time-restricted eating or intermittent fasting. Furthermore, walking-based exercise interventions are well-tolerated [35, 36], especially by those with lesser functional/cardiorespiratory capacity [37]. Therefore, we designed a free-living, remotely monitored, walking-based exercise intervention, with the primary aim of assessing the feasibility and acceptability of manipulating the timing of each exercise session relative to breakfast, in people living with overweight or obesity. The secondary aims were to determine whether completing each exercise session either before or after breakfast would differentially affect glycaemic control and other markers of cardio-metabolic health.

## Methods

### Participant information

Thirty-four sedentary overweight individuals were recruited from all areas of the UK via social media (see Table 1 for characteristics). They were deemed sedentary if they performed less than two 30-minute structured exercise sessions per week and therefore would not meet the minimum recommended amount of physicial activity [38]. We excluded those people taking any glucose-lowering or weight loss medication. Participants provided written informed consent, and the study was approved by the Liverpool John Moores University Research Ethics Committee (UREC– 20/SPS/021) and conformed to the Declaration of Helsinki. This study began recruitment and data collection between 1^st^ May 2020 and 30^th^ April 2021 at the height of the COVID-19 global pandemic lockdown. Thus, all data collection and the delivery and undertaking of the exercise intervention was conducted remotely, using equipment and tracking services that were available, or made available to the participant themselves, thereby minimising the risk of virus transmission. As standard practice in our institute, all participants completed a readiness to exercise questionnaire to identify any medical issues that would preclude safe participation in the study.

**Table 1:** Subject characteristics, and changes in anthropometric variables and fitness in response to the exercise programme in both FASTED and FED groups.

Variable	FASTED		FED		Time effect		Time x group	
	Baseline	Follow-up	Baseline	Follow-up	(p value)	(p value)	
***n* (M/F)**	7/10		5/10		-	-	
**Age (years)**	47 ± 11		43±12		-	-	
**Height (m)**	1.71 ± 6.8		1.69±9.8		-	-	
**Weight (kg)**	101.3±17.1 (83.0–130.6)	97.5 ± 15.9 (78.5–130)	98.5 ± 20.5 68.9–140.0)	95.4 ± 21.0 (64.4–134.4)	**<0.001**	0.620	
**BMI (kg.m** ^**-2**^ **)**	34.8 ± 5.1 (29–43.8)	33.6 ± 5.0 (25.9–42.5)	34.1 ± 4.8 (27.6–43.8)	33.1 ± 4.9 (25.8–41.5)	**<0.001**	0.369	
**Waist circumference (cm)**	110 ± 16 (90–142)	103 ± 16 (83–138)	108 ± 17 (92–140)	103 ± 16 (86–131)	**<0.001**	0.812	
**Hip circumference (cm)**	117 ± 8 (105–132)	111 ± 8 (95–129)	116 ± 11 (100–137)	110 ± 11 (96–133)	**<0.001**	0.868	
**Waist:hip ratio**	0.94 ± 0.13 (0.76–1.24)	0.93 ± 0.12 (0.8–1.1)	0.94 ± 0.11 (0.7–1.1)	0.93 ± 0.10 (0.82–1.20)	0.290	0.871	
**Fitness Index**	98 ± 21(68–149)	110 ± 17 (86–142)	98 ± 20 (63–144)	108 ± 15 (74–132)	<0.001	0.662	

Data are presented as means ± S.D with the range presented in brackets below.

### Experimental protocol

Using a parallel groups design, participants took part in a 12-week walking-based exercise programme where each prescribed exercise session was undertaken either before (FASTED) or after breakfast (FED). Participants were randomised upon recruitment in an alternating group allocation approach whereby participants 1, 3, 5 etc were allocated to FED, and participants 2, 4, 6 etc were allocated to FASTED. The lead researcher was not blinded to the group allocations. Before and after the intervention, we measured anthropometric data, cardio-respiratory fitness, plasma biochemistry (seum lipids and liver transaminases) and free-living glycaemia. Participants received a study pack by post which contained a Bluetooth heart rate (HR) monitor (either a Polar H10 chest strap HR monitor or a Polar Verity Sense optical HR sensor), two Freestyle Libre continuous glucose monitors (Abbott, UK), a fabric tape measure, and an instruction booklet containing a timetable for the study, details of each assessment and the outline for the training programme. A commercially available capillary blood sampling kit (Thriva, UK) was delivered directly by post to the participant. The Freestyle Libre CGM has been reported to have acceptable clinical accuracy [39], and the Thriva blood sampling kit demonstrates good clinical utility [40]. Before undertaking any baseline assessments, all participants had a video call with the lead researcher (JB) to talk through the assessments and answer any questions.

**Fig 1:**
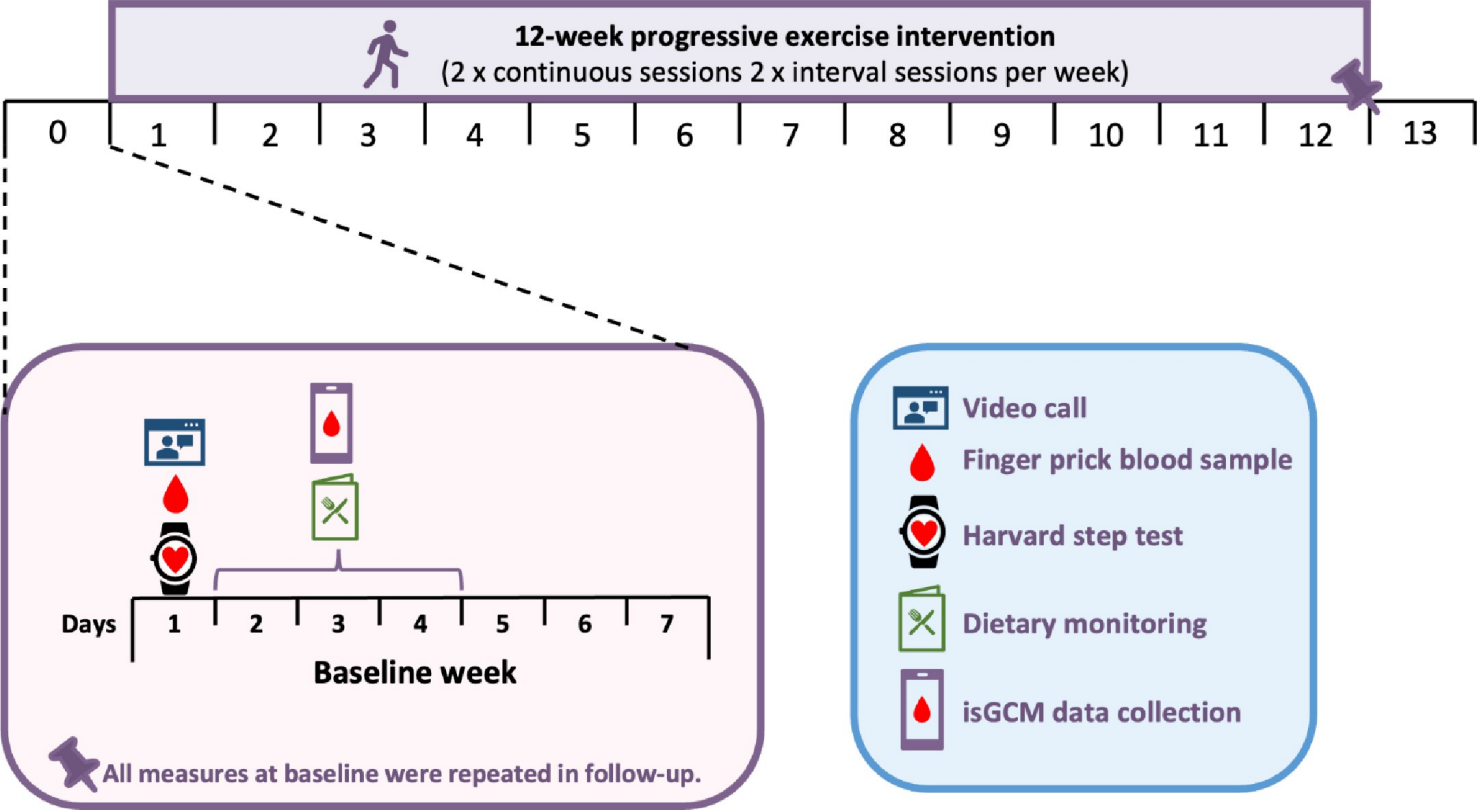
Overview of study protocol. **Week 1–2** Individuals inserted the continuous glucose monitoring (isCGM) on day 1 of baseline week for two weeks to include the first week of intervention). During the baseline week, participants carried out a finger prick blood sample, a fitness test (day 5 or 6) and kept a food diary for 3 days. Data from the isCGM was analysed from the 3-days a food diary was kept. **Week 12** An isCGM was inserted at start of week 12 of the intervention and this remained in place for two weeks (i.e., week 12 of intervention and follow up week). In follow-up week, participants repeated the finger prick sample, fitness test and reproduced their dietary intake of the 3 days in the baseline week.

On day 1 of the baseline week, participants collected a fasting capillary blood sample using a commercially available home blood testing kit (Thriva, UK) for the measurement of blood lipids, markers of liver function and HbA1c, recorded their own body weight, waist and hip circumference measures, and performed a fitness test (Harvard step test) (see S1 File). These assessments were repeated on the first day of the follow-up week (Fig 1). An intermittently scanned continuous glucose monitor (isCGM) was also inserted on day 1 and remained in place for 2 weeks (i.e. baseline week and week 1 of the intervention). This was repeated at the beginning of week 12 and remained in place for 2 weeks, such that isCGM glucose data was collected 7 days post-intervention. Only the data collected during the baseline and follow-up weeks are reported in this manuscript. The sensor was inserted on the upper arm using the instructions provided on the Freestyle Libre app and supervised by a researcher on a video call. Following a 24 h ‘bedding in’ period, data was collected from day 2 to day 4 during the baseline week (Fig 1). The post-prandial responses to each meal, defined as AUC for the 3 hours following the time participants recorded as the time of each meal, was measured. At the same time, diet was recorded in a self-reported food diary for the pre-intervention assessment week to capture habitual diet as well as diet during the 3 specific days of CGM analysis. Participants were sent their food diary from the beginning of the intervention and were asked to consume an identical diet for 3 days in the follow-up week (Fig 1).

### Exercise and diet intervention

The exercise intervention was identical in all respects for all participants, only differing by the nutritional status under which the exercise sessions were completed (detailed below). The intervention included four exercise sessions per week for 12-weeks, incorporating two continuous walking sessions and two interval walking sessions. A combination of continuous and walking exercise sessions was chosen considering both types of exercise have previously been reported to be effective for improving markers of cardio-metabolic health in individuals at risk of type 2 diabetes [25, 41]. The continuous session required participants to walk at a pace that maintained their HR between 50 and 60% of estimated HR maximum (HR_max_; calculated as 220 minus age). The interval sessions required participants to alternate between 3 minutes of walking at 50–60% HR_max_ and 3 minutes at 80–90% HR_max_. All exercise sessions in week 1 were 30 minutes in duration and increased every two weeks by 5 minutes for the continuous walking sessions and 6 minutes for the interval walking sessions based on previous studies from our lab [42]. Participants in FAST were instructed to complete their prescribed exercise session before consuming anything other than water and consume their habitual breakfast within 30–90 minutes of completing the exercise session. In FED, participants were required to consume their habitual breakfast, and then complete their prescribed exercise session 30–90 minutes later. Participants had a weekly video call with the lead researcher to check-in.

### Training monitoring and exercise data analysis

All participants were provided either a Polar H10 chest strap heart rate monitor or a Polar Verity Sense optical heart rate sensor to have real-time HR readings during the exercise sessions to help them meet the desired exercise intensity for the sessions. The HR data was also used to assess adherence and compliance to the exercise programme. All HR data from each exercise session was stored on a cloud-based system, available at www.flow.polar.com. For each continuous walking session, data pertaining to exercise duration and mean HR (as a % of HR_max_) was extracted. For each interval session, the number of intervals completed, peak HR on each interval, % of intervals achieving the criterion HR (≥80% HR_max_), and time spent above the criterion HR were recorded. Adherence was calculated as the % of prescribed sessions completed during the programme. Compliance to the prescribed exercise is defined differently for continuous exercise sessions and interval sessions, and has previously been detailed by Hesketh et al., (2021), but generally refers to the achievement of both a prescribed duration and intensity. For the continuous exercise sessions, duration was adjusted for the exercise intensity to produce a HR physical activity score (HRPAS = min × % HR_max_) for each session [43]. If the session HRPAS was equal to or greater than the prescribed HRPAS, the session was compliant. Interval session compliance was defined as achieving a HR ≥80% HR_max_ during the session and performing the prescribed number of intervals.

### Dietary analysis

**Fig 2:**
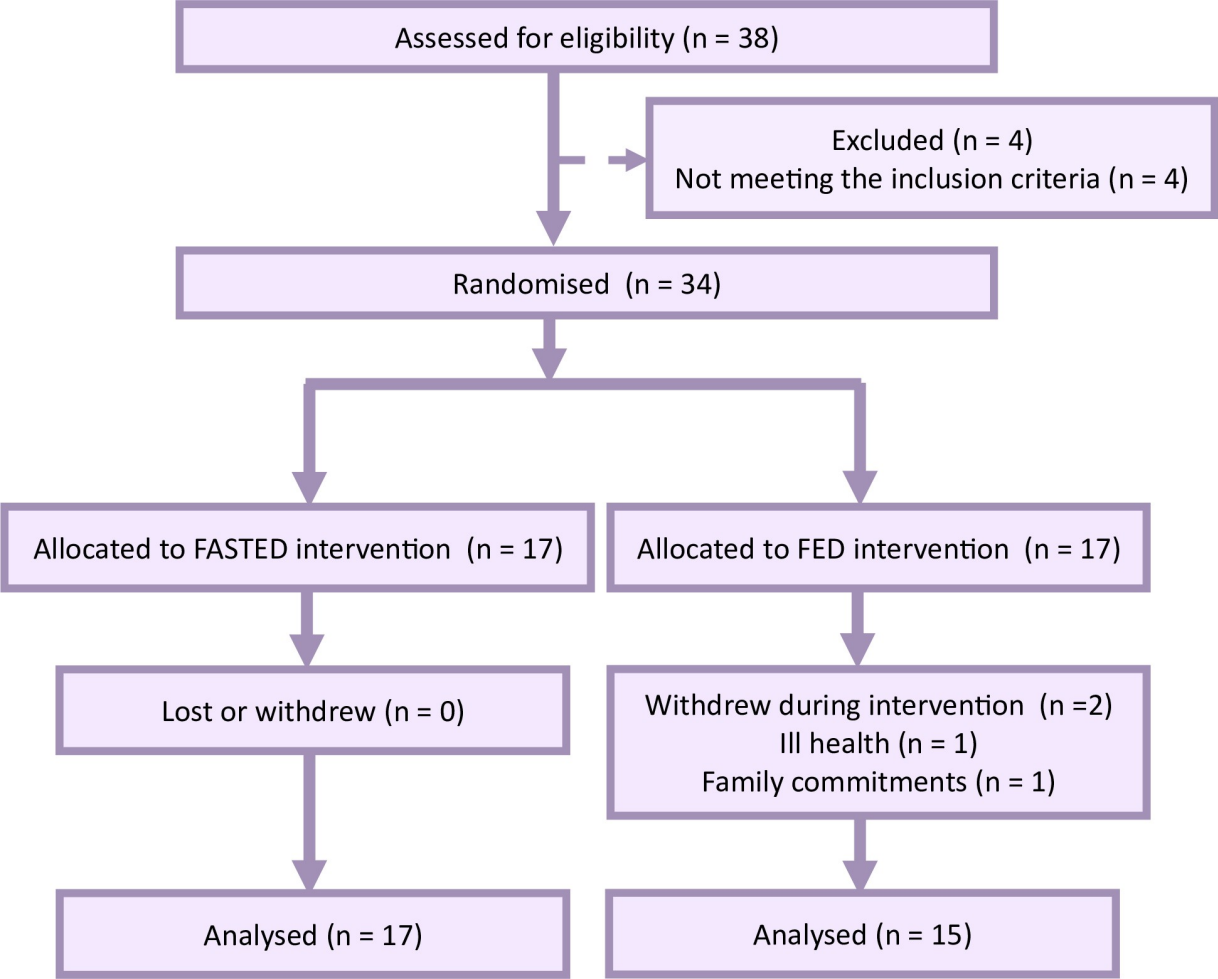
Trial flow diagram. The progress through the phases of a parallel randomised trial of two groups including enrolment, intervention allocation, follow-up, and data analysis.

Participants were asked to record their diet during the 3 pre-intervention days in which CGM measures were taken, and then repeat this diet during the post-intervention.CGM data collection. Separate to this, participants were also asked to record their food and beverage intake for 3 days pre- and post-intervention to enable a comparison to be made and attempt to understand whether habitual dietary intake may have changed (Fig 2B). Diaries were analysed using Nutritics software (Nutritics Ltd., Co. Dublin, Ireland) for total energy (kcal) and macronutrient intake. All analyses were carried out by a single trained researcher to minimise any potential variation in data interpretation.

### Focus group

One focus group was conducted (by JB) with study participants (*n* = 6, 3/3 F/M) via Zoom video conferencing with audio-visual recording. A random group of participants from our participant pool were contacted (counter-balanced between the two conditions; fed versus fasted) to form our focus group. The aim of the focus group was to gain insight into the facilitators and challenges to taking part in this free-living exercise intervention. The focus group session (~45 minutes) sought clarification of information during the questioning process to ensure participants were able to expand on each-others opinion and summarise the information provided [44, 45]. The focus group was transcribed verbatim, and data were thematically analysed manually using reflexive thematic analysis. Recommendations such as data familiarisation, generating initial themes, coding and finalising patterns of shared meanings underpinned by a central concept, and writing up using data extracts interspersed with researcher interpretations [46].

### Statistical analysis

A formal power calculation was performed (G*Power 3.1, Kiel, Germany) to determine the required sample size. Because no data was available for the HbA1c response to fed versus fasted exercise training, we used the data from a training study in healthy, lean males which compared the effects of fasted versus fed-state exercise on glycaemic control insulin sensitivity [47]. In that study, a change in the plasma glucose area under the curve for an oral glucose tolerance test mean [SD] of –13 [12] mmol·min·L^-1^ was observed in the fed training group, whereas a mean [SD] of -65 [63] mmol·min·L^-1^ was observed in the fasted training group. Using the calculated effect size (*d* = 1.14), and α set at 0.05, 28 participants were required to detect a significant difference in insulin sensitivity with 85% power. Allowing for a drop-out rate of 15%, we recruited 17 participants per group. All data were analysed using statistical analysis software (Statistical Package for the Social Sciences for Windows, version 27.0, Chicago, IL, USA). A mixed-design ANOVA was used to interrogate all time and group-dependent data, whereas an independent t-test was used to investigate differences in adherence and compliance to the exercise intervention between FAST and FED. Data are presented as means ± S.D. Significance was set at *P*<0.05.

## Results

### Adherence and compliance to exercise sessions

Two participants in FED withdrew their participation (Fig 2), meaning the data presented includes 17 individuals in FASTED and 15 individuals in FED. There were no adverse clinical events during the study. The mean duration and HR responses to the exercise sessions over the 12-week programme are reported in S1 and S2 Tables in S2 File. Overall adherence to the exercise programme was similar between conditions (*P* = 0.456), with 94% and 95% sessions completed in the FASTED and FED groups, respectively. Considering each type of exercise session, similar rates of adherence were observed between groups to both the continuous sessions (FASTED 95±10%; FED 96±5%; *P* = 0.656) and interval sessions (FASTED 93±7%; FED 95±6%; *P* = 0.639).

Compliance to the continuous walking sessions was not different between groups (FAST 84±13%, FED 85±12%; *P* = 0.955). Interval session compliance was defined as achieving a HR ≥80% HRmax during the intervals in the session and performing the prescribed number of intervals, and again, compliance to the interval walking sessions was similar between groups (FAST 86±11%, FED 90±13%; *P* = 0.671). Thus, total compliance to all the exercise sessions was 85% for FAST and 87% for FED.

### Anthropometric data

Baseline characteristics are reported in Table 1. At baseline, there were no significant differences between the two groups for age or any anthropometric variables. Body mass, (-3.8 kg in FASTED, -3.1 kg FED; *P* <0.001), BMI (*P* <0.001), and waist and hip circumferences (-7 cm in FASTED, -5 cm FED; *P* <0.001) were decreased from pre- to post-intervention, and fitness index (*P* <0.001) increased over time, with no difference between the two groups (*P*>0.05).

### Energy intake and macronutrient composition

Total energy intake and the macronutrient composition (in both absolute and relative terms) of each meal was similar across the three days during baseline and follow-up (S3 Table in S2 File). Dietary data was also collected on the prescribed exercise days during week 1 and week 12 of the intervention. Total energy and macronutrient intake for breakfast, lunch and dinner were analysed in the 3-day food diaries at baseline and follow-up. Total energy, carbohydrate, protein and fat intake at breakfast, lunch and dinner was not statistically different at baseline compared to follow-up (*P* > 0.05).

### Blood lipids, HbA1c, and markers of liver function

Blood lipids, HbA1c, and liver function tests are reported in Table 2. At baseline, the two groups were well matched clinically. Twelve weeks of walking exercise training did not alter total cholesterol, triglyceride, LDL cholesterol or HDL cholesterol concentrations in either group (*P*>0.05). Importantly though, the 12-week training intervention significantly reduced HbA1c (*P* = 0.01), although the magnitude of the reduction was relatively small and similar between conditions (*P*>0.05); the mean change in HbA1c for FAST was -3 mmol/mol (ranging from -4% to +1%), and 1.1 mmol/mol (ranging from -7% to +2%) in the FED group. The training intervention also had a significant effect on serum ALT concentrations, with a decrease being observed following training (*P* = 0.006). Post-hoc analysis revealed the decrease was specific to FAST (- 5.5 UI/L, *P* = 0.001) but was not apparent in FED (-0.5 UI/L, *P* = 0.720). For all other liver biochemical measures, there was no significant changes following training (see Table 2).

**Table 2:** Changes in HbA1c, blood lipids, and markers of liver function in response to FAST or FED training.

Variable	FASTED			FED			Time effect	Time x group
	Pre		Post	Pre		Post	(p value)	(p value)
**Glycaemia**								
HbA1c (mmol/mol)	38±4	35±4		37±5	36±4		0.010	0.763
**Lipid profile**								
Triglycerides (mmol.L^-1^)	1.73±0.6	1.70±0.6		2.03±1.0	1.82±0.7		0.343	0.498
Cholesterol (mmol.L^-1^)	5.7±0.8	5.6±0.8		5.6±1.1	5.5±1.0		0.440	0.909
LDL-cholesterol (mmol.L^-1^)	3.5±0.6	3.5±0.7		3.3±0.9	3.3±0.8		0.783	0.672
HDL-cholesterol (mmol.L^-1^)	1.4±0.3	1.4±0.3		1.3±0.2	1.3±0.2		0.243	0.611
**Liver biochemistry**								
Gamma-GT (IU/lL)	36.4±6.53	34.3±11.45		57.5±10.93	58.3±9.16		0.779	0.566
Alanine Aminiotransferase, ALT (IU/L)	33.9±17.5	28.4±14.9*		28.3±19.6	27.8±20.4		**0.006**	**0.021**
Albumin (g/L)	41.5±2.4	40.9±2.2		42.6±2.4	42.4±2.0		0.294	0.576
Alkaline Phophatase (IU/L)	83.0±25.9	87.2±30.0		81.7±21.7	80.8±22.0		0.374	0.180
Globulin (g/L)	30.2±3.3	30.4±3.1		30.7±4.2	30.9±3.4		0.623	0.919
Bilirubin (umol/L)	10.5±4.0	9.5±3.3		9.7±2.3	10.1±2.5		0.504	0.178
Total Protein–(g/L)	72±4	67±16		73±6	73±5		0.234	0.124

Data are mean ± SD, significance *P*<0.05. Post hoc analysis indicates significant change post intervention in the FAST group only (*). Total protein refers to (albumin & bilirubin combined).

### Glycaemic control at baseline and follow-up

Mean glucose over the 3-days of recorded diet at the start of the intervention was 6.0 ± 1.0 mmol·L^-1^ for FASTED and 6.1 ± 1.5 mmol·L^-1^ for FED (P = 0.295). The coefficient of variation for the mean glucose over the 3-days at baseline was 10 ± 4% for FASTED and 14 ± 8% for FED with no difference between groups (*P>*0.05). Following the intervention mean glucose tended to be reduced in FAST (5.8 ± 1.2 mmol·L^-1^; *P* = 0.09) but was unchanged in FED (6.2 ± 1.4 mmol·L^-1^; *P =* 0.90). The intervention did not alter the glucose coefficient of variation in either FASTED(12% ± 9) or FED (11% ± 4; *P*>0.05).

**Fig 3:**
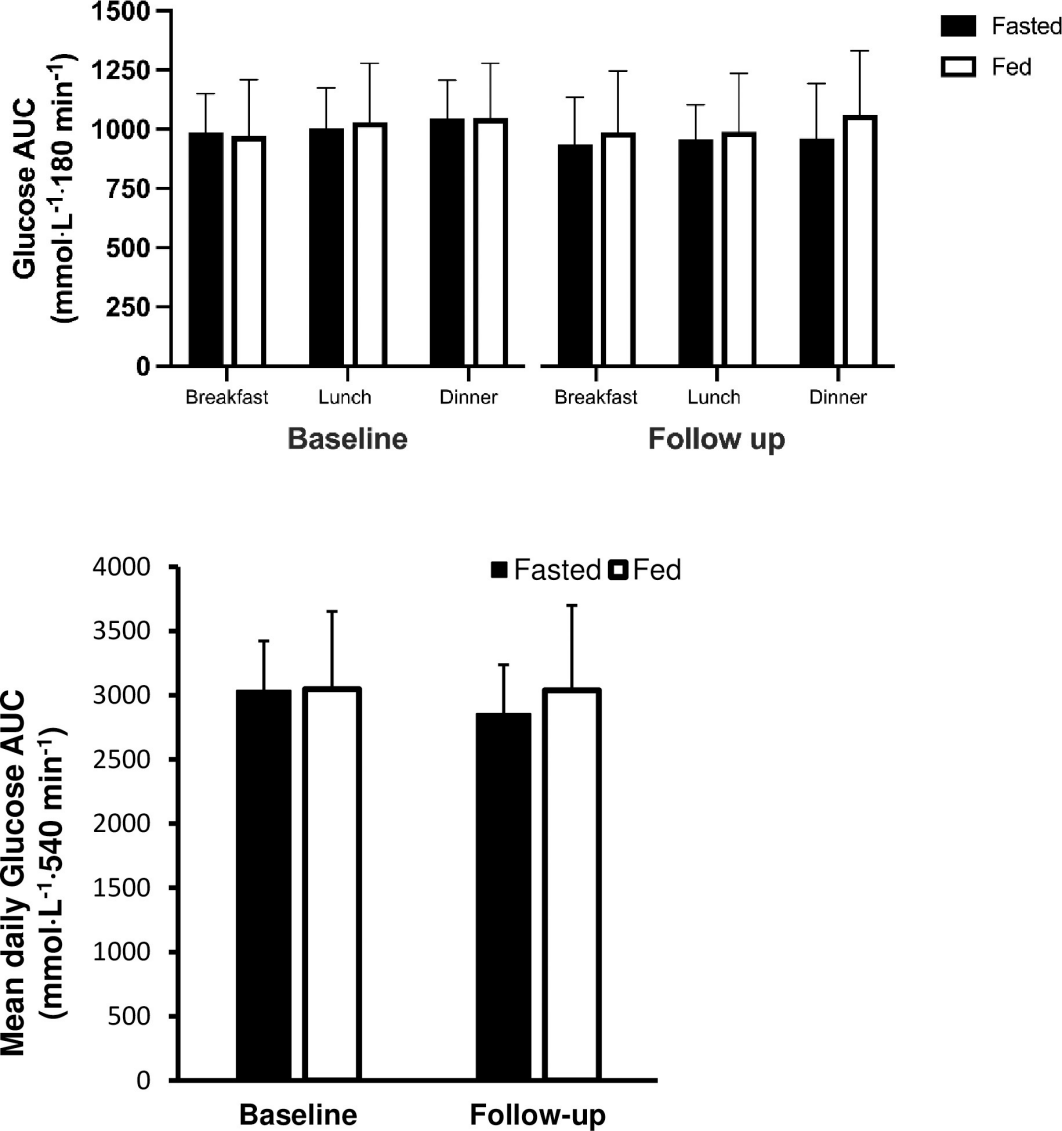
Area under the curve responses to breakfast, lunch and dinner during the baseline week and follow-up week. AUC was significantly greater following dinner (*) compared to following breakfast (*P >*0.05). Data are mean ± SD.

The postprandial responses to each meal, defined as AUC for the 3 hours following the time participants recorded as the time of each meal, were also examined (see Fig 3). At baseline, there was a main effect of meal detected (*P* = 0.040), whereby glucose AUC was significantly greater following dinner compared to breakfast (*P* = 0.047), which corresponded to mean glucose also being greater following dinner compared to breakfast (*P* = 0.020). We report no significant difference in total daily glucose AUC (*P =* 0.211; see Fig 3B).

When examining post-prandial glucose responses at baseline compared to follow-up, there was a significant time × meal interaction (main effect; *P* = 0.047). However, the post hoc analysis revealed no significant changes in postprandial glucose AUC to any of the three meals from baseline to follow-up. Rather, there was only a difference in glucose AUC in response to breakfast versus dinner prior to the training intervention (post-hoc analysis of time × meal effect; *P =* 0.046), whereas following the intervention this meal-specific difference disappeared (post-hoc analysis of time × meal effect; *P =* 0.135). Peak glucose during the postprandial period was greater following dinner compared to breakfast (main meal effect; *P* = 0.043), with no difference between groups or from baseline to follow-up (*P >*0.05).

### Focus group findings

Table 3 illustrates the themes and sub-themes identified during the analysis and is supported by verbatim quotes. There were three themes for facilitators; exercise monitoring; structure; blood testing; and two themes for barriers/challenges to completing the exercise programme; time of year and time. Importantly, these barriers highlight challenges that individuals faced but did not result in reductions in adherence and compliance to our programme. Table 3 also highlights our two themes that emerged for support in behaviour changes; habit development and using technology to increase exercise levels.

**Table 3:** Facilitators, challenges and behaviour change support of the exercise-nutrition intervention.

Facilitators/Challenges	Sub-theme	Illustrative quote
**Facilitators**	**Exercise monitoring/ Structure**	I like the way you designed the exercise, what is good is you control your heartbeat and that’s really useful, so I found that really good… you know what you need to do, when you need to speed up to help you or just a slow down so that is really good to follow the structure (participant 1).
		I thought (the programme) had a clear structure what was happening at each stage so obviously the information was very useful, but I think you explained at every stage and then you could sort of see the progress, so you know getting a bit fitter (participant 4).
	**Support**	You checking in with us as well like just seeing how we’re getting on and just asking questions and stuff and yeah cause I think I started in lockdown happens and I was like how am I going to do this nearby and you were just in touch and I do think part of the success is due to this (participant 4).
	**Blood testing**	I like getting the result after the old blood test that is really good that is really helpful (participant 3).
**Challenges**	**Time of year**	When it was raining outside. Yes, the weather made it hard (participant 3).
	**Time**	If we weren’t in lockdown the time would have been difficult time commitment (participant 2).
**Support in behaviour changes**	**Habit development**	It motivated me…since I’ve left I’ve lost a stone and ½…(the programme was an) initial kick up the backside that was great for me (participant 5).
	**Using technology to increase exercise levels**	I did the app 100,000 steps challenge in whatever month it was but again I wouldn’t have monitored my fitness like that unless I had got the experience of doing it for you (participant 4).

### Facilitators

#### Exercise monitoring/structure

All participants agreed that being able to monitor your exercise sessions using the HR monitor helped to complete the exercise sessions. It was evident that having guidance and ‘target zones’ to aim for each of the exercise sessions helped encourage the exercise sessions to be completed and took away that aspect of uncertainty around exercising ‘correctly’ that individuals often feel deters them from completing exercise.

*“I do like the way you designed the exercise. One reason I liked it is that I need to go outside to walk that is pretty good and as everybody is saying you’re motivated to get out the house you’re doing exercise especially if you go outside so you get fresh air so that part, I find really useful and what is good is you control your heart beat so that’s really useful” (participant 1).*

#### Support

Participants stated that having regular weekly ‘check-in’ calls with the researcher was a significant contributor to the success of the intervention my enabling the opportunity to feel supported and ask questions, eg., “*You were just in touch*, *and I do think part of the success is due to yourself and being very friendly*” (participant 3).

#### Blood testing

It was evident that all participants felt the blood test they completed before and after the exercise programme provided both a reason to first sign up to the programme but also a strong motivator to complete the exercise programme to try and improve these values as identified by participant 4; *“Well*, *the best part for me really so one of the good thing was that I found out my cholesterol level was high and… I’ve managed to get it right down” (participant 5)*.

### Challenges

#### Time

Since this intervention was completed during the COVID-19 global pandemic, individuals found it possible to do these exercise sessions either before or after breakfast. Those who we’re in the FED group and needed to eat their breakfast and wait 60 minutes before going out to do their exercise session said that this would not be possible if they needed to be in work like normal, eg., *“To do the walk and get to work in time I think that we’d find that impossible” (participant 2)*.

### Support in behaviour change

#### Habit development

It was evident that the programme resulted in a positive behaviour change whereby all participants that were involved in the focus group agreed that they continued to exercise following the end of the program. Participant 3 identified this and the positive effects it had when they said; “After the study I walk every day and weekends and I haven’t done that before so that basically changes in everything in my life”. Participant 2 also identified that the blood test encouraged a further behaviour change by saying; “*we`re more physically active but also*, *we’ve both given up alcohol because of what the (blood) tests were showing*.

#### Using technology to monitor exercise

Participants were positive about the influence that technology had during exercise and that they continued to use it after the end of the programme; *“using the app…it’s something I’ve never done in my exercise*, *so now I do this quite often I use the app to do more exercise (participant 2)”*.

## Discussion

The primary aim of the current study was to determine the feasibility of manipulating the timing of exercise relative to breakfast during a free-living walking-based exercise programme in individuals living with overweight or obesity. First, we demonstrate that this walking-based intervention, involving both continuous and interval walking exercise sessions, was associated with high adherence and compliance for sedentary individuals living with overweight and obesity. Secondly, we investigated whether this walking-based intervention has differential benefits to glycaemic control and markers of cardio-metabolic health whether undertaken before or after breakfast. Although there was a decrease in body mass and HbA1c concentrations from pre- to post-intervention, we did not observe significant improvements in glycaemic control following the intervention for either group. However, exercising in the fasted (vs, fed) state seemed to induce greater reductions in surrogate biochemical markers of NAFLD, suggesting potential improvements in liver health. This study serves as preliminary data for a larger follow-up randomised control trial.

### Suitability of the programme for individuals with overweight and obesity

Whilst the free-living nature of the programme was chosen due to the COVID-19 pandemic, this provided the opportunity to develop and test whether our exercise intervention could be successfully implemented into the daily lives of individuals living with overweight and obesity. Here, we show that this 12-week combined continuous and interval walking intervention had high adherence in which participants completed ~96% of the prescribed exercise sessions with a high level of compliance (~86%). Previous studies which aimed to implement free-living exercise interventions in sedentary individuals living with overweight and obesity generally report low rates of adherence, with only 39–48% [48], 36–67% [49] and 25–30% [50] of prescribed exercise sessions actually completed. When compared to these studies, we report remarkably high rates of adherence to our exercise programme, and importantly, the adherence to the programme was similar whether individuals were randomised to exercise before or after breakfast.

Research in individuals with T2D show that where interventions include supervised exercise sessions, they also increase total physical activity levels [51]. However, the resources (e.g., cost of exercise practitioners, time needed to travel to facilities, physical space for exercise sessions) for the implementation of such interventions are not feasible in many countries [52]. Supervision of exercise sessions facilitates motivation, a major barrier to physical activity in individuals living with overweight/obesity [53–57]. In the current study, all participants received a video call once per week from the lead researcher (JB). Feedback from participants at the end of the study showed that the regular contact with the lead researcher provided them with the motivation to continue with the programme, as well as acting as a form of accountability. Knowing the researcher could check their exercise sessions each week, since they were recorded on the HR monitor and uploaded to a cloud-based system, also likely contributed to the sustained motivation and adherence to the programme. Participants felt that having a heart rate monitor alongside target HR zones for each walking session gave them a structure to follow. These observations are in line with a recent study from our laboratory showing that the same HR monitoring system was successful in improving adherence to structured home-based exercise programmes in individuals with obesity and an elevated cardiovascular disease risk [58]. Together, this study and observations from the current work demonstrate that weekly contact with an exercise physiologist paired with a simple form of wearable technology can result in high rates of adherence and compliance to a free-living exercise intervention.

### Effect of the programme on fitness, anthropometric measurements and routine biochemical markers

In line with the well-known effects of exercise on aerobic fitness, it was no surprise that both groups observed similar improvements in their cardio-respiratory fitness index score as assessed using the Havard step test. This is an important observation given the strong link between poor cardio-respiratory fitness and greater cardiovascular disease risk [59]. Likely due to high levels of adherence to the programme, we also observed a significant decrease in body weight (~ 3kg reduction) after the 12-week exercise intervention, in line with previous studies employing similar interventions in similar cohorts [60, 61].The magnitude of improvements in weight are different for each co-morbidity but clinically significant weight loss is defined as at least a 5% reduction in body mass [62] which neither group quite achieved (FASTED; 4%; FED 3%). We do, however, report a clinically significant reduction in waist circumference (~7 cm reduction), hip circumference (~6cm reduction) and waist-to-hip ratio, considering the strong association with all-cause [63, 64] and cardiovascular mortality [65–67], a 7 cm (6.0%) decrease in waist circumference in the FAST group and a 5 cm (4.6%) decrease in the FED group. Participants did measure this themselves, so there is potential for greater measurement error, but we limited this by specifying the exact distance participants should place the tape measure above the belly button. A reduction of 3 to 6.8 cm in waist circumference is considered clinically relevant [67], meaning that over the 12-weeks of either fasted or fed exercise training, we saw a clinically relevant reduction in waist circumference.

HbA1c values were reduced by a small, but significant, value of ~3±4 mmol/mol in FASTED and ~1±4 mmol/mol in FED (~7% reduction in the FAST group and 2% reduction in the FED groups). It appears that the walking exercise intervention alone, independent of the nutritional state in which exercise was undertaken, was sufficient to increase glucose regulation. These data differ from data in individuals with T2D [16] which reported minor and non-significant changes in HbA1c with a similar intervention. Using isCGM in the present study, we didn’t observe any change in free-living postprandial glucose responses to daily meals, or any markers of glycaemic variability, in response to the exercise programme in either group. The free-living nature of our study design may have contributed to this observation, although it is important to note that on CGM data collection days at baseline and follow-up, we specifically asked participants to consume an identical diet. In a more controlled study investigating the effect of six weeks moderate intensity cycling in overweight/obese males undertaken in the fasted or fed state, there was also no improvement in glucose control during an oral glucose tolerance test [32]. Overall, it remains to be determined whether exercise-nutrient interactions are an important modulator of short-term glycaemic variability and a reduction in HbA1c in the longer-term. It is important to consider that consuming carbohydrate 30 to 45 minutes before exercise can result in a rebound hypoglycaemia in some individuals [68]. During the weekly check-ins throughout the study, none of the participants reported any symptoms of hypoglycaemia (e.g. light-headedness) during their training sessions. This would be important to objectively monitor in future studies as it could influence ongoing adherence to the intervention.

Regular physical activity plays an important role in preventing or treating metabolic dysfunction-associated steatotic liver disease (MASLD) [69]. Exercise is known to reduce systemic inflammation and oxidative stress in MASLD and is inversely associated with hepatic-fibro-inflammation, particularly at greater intensities, when measured by quantitative MRI [70]. Therefore, we included surrogate markers of liver health in our biochemical analysis. Of liver biochemistry, only ALT decreased following the 12-week exercise intervention (in the fasted but not fed exercise group). ALT is not a direct measure of liver fat and can underestimate liver fat in individuals living with T2D [71]. Furthermore, a recent systematic review and meta-analysis highlighted that exercise interventions reduce liver fat in the absence of significant weight change, and thus meaningful reductions in liver fat may not be reflected in serum ALT concentrations [72]. However, it is known that the degree of weight loss correlates with improvements in histological measures of MASLD [73]. Our data suggest that an additional nutritional component to the intervention is required to induce a reduction in ALT concentrations. Indeed, we observed a 16% (vs 2%) reduction in ALT concentrations in the fasted group (versus fed) group, and only the fasted exercise group experienced a significant reduction in ALT concentrations. Exercising in the fasted state leads to greater mobilisation of fat stored in subcutaneous and visceral adipose tissue [74, 75] and contribution of fat to energy expenditure. Therefore, it could be suggested that during an intervention where exercise is repeatedly undertaken in the fasted state that there will be increased whole-body lipid turnover, including in tissues such as the liver. Consequently, this could contribute to improving liver health, recognised as a decrease in ALT concentrations. Whether the intervention would be associated with a significant reduction in liver fat requires further investigation using MRI assessment [76]. It is also important to note that while none of our participants had received a diagnosis of MASLD prior to entering the study, the baseline blood tests showed that 8 individuals had elevated ALT concentrations (>50 IU/L); 5 in the FASTED group and 3 in the FED group. Importantly, all 8 of these individuals experienced a reduction in ALT concentrations following the intervention, and 4 of those 8 (2 in each group) were within the normal range (15–50 IU/L) at the follow-up blood test. Thus, the exercise programme was effective in reducing ALT concentrations in those most at risk of, or who have pre-existing MASLD.

Meta-analyses reveal that walking-based interventions are effective at improving aerobic capacity, insulin sensitivity and other cardiovascular disease risk factors such as blood pressure and waist circumference, but often fail to significantly improve serum lipid profiles [25, 41]. In agreement with these meta-analyses, we did not observe any significant changes to the serum lipid profile (triglycerides, cholesterol, HDL and LDL) from baseline to follow-up in either group. This is in agreement with a recent study comparing exercise training in the fed or fasted state in individuals with T2D, which also failed to improve total cholesterol, LDL and triglyceride concentrations [16]. It is possible that caloric restriction is required to improve the lipid profile [77] and that changes in apolipoproteins and lipoprotein particle size might be improved independent of lipid levels [78]; both factors should be considered for future walking-based exercise programmes aiming to reduce cardiovascular disease risk.

### Strengths and limitations

This study was completed during the COVID-19 global pandemic, with the exercise programme and all outcome measures completed without researcher-participant contact. Despite this, we observed significant improvements in HbA1c, anthropometric measurements, and in ALT concentrations (in individuals who exercised fasted). Therefore, our exercise intervention could be implemented in communities to facilitate weight change and diabetes prevention. One limitation of this study is the lack of a non-exercising control group meaning that drawing causal inferences for the effect of exercise is inappropriate. However, this does not limit our ability to derive inferences regarding the effects of exercise before compared to after breakfast. Secondly, our method of group allocation meant that each participant did not have an equal chance of being assigned to each intervention [79]. Since our primary aim was to perform a feasibility study (rather than estimating causal effect) this randomisation strategy is not a major issue, but we do acknowledge that using a more appropriate randomisation approach would be important for future larger-scale trials. We acknowledge that the HR formula used to estimate training zones has a high degree of inter-individual variability, but this is what would be used in the real world and therefore represents a useful starting point for all participants. We acknowledge that it isn’t the perfect approach for determine training zones, but under the current circumstances was entirely appropriate. We did not monitor dietary intake during the intervention, and at best, the food diary analysis pre- and post-intervention serves as a measure of whether changes in habitual diet occurred in response to the intervention. Lastly, physical activity levels were not measured objectively (e.g. using accelerometery) but given that the government guidelines regarding lock-down were in force during the study we can assume that physical activity level was relatively stable.

## Conclusion

The current study provides evidence that a free-living walking-based exercise intervention where exercise is timed relative to breakfast can be successfully implemented into the lives of people living with overweight and obesity. We show significant benefits to several health markers in response to the exercise programme irrespective of the nutritional status under which exercise was undertaken. However, walking before breakfast lead to greater reductions in serum ALT concentrations (a surrogate measure of liver fat accumulation). Future imaging-based studies could determine whether physical activity, in a fasted state, enhances the therapeutic benefits of exercise for liver health.

## Supporting information

S1 File Extended methods. (DOCX)

S2 File (DOCX)
