# Supplementary material for: A free-living, walking-based, exercise programme, with exercise timed relative to breakfast, to improve metabolic health in people living with overweight and obesity: A feasibility study
Source: PLoS One. 2024 Nov 21;19(11):e0307582. doi: 10.1371/journal.pone.0307582 (PMC11581328; doi:10.1371/journal.pone.0307582)
Supplement: S1 File — (DOCX) [file pone.0307582.s001.docx]

Supplementary Material 1:

**Methods**

*Body Composition*

Participants were provided with a tape measure and scales (if required), to measure their height (cm), weight (kg), waist and hip circumference (cm). The instruction booklet provided in the study pack contained details for the correct area to measure waist and hip circumferences. These values were then used to calculate waist-to-hip ratio.

*Fitness test*

Participants were asked to carry out the Harvard step test as a measure of fitness (20). Briefly, participants were required to step onto, and back down from a step ~30cm high at a rate of 30 completed steps per minute (one second up, one second down) for 5 minutes. The individuals immediately sat-down following completion of the test, and the total number of heart beats were counted from 1 to 1½ minutes after finishing, from 2 to 2½ minutes after finishing, and finally from 3 to 3½ minutes after finishing.  The participants wore the HR monitor during the test to record beats per minute, and from this, a ‘fitness index’ measure was calculated using the following equation:

Fitness index = 30000 ÷ (pulse1 + pulse2 + pulse3)

The fitness index was then compared to normative data available for 16-year-old athletes (Beashel and Taylor, 1997). No normative data for our population is currently available.

*Blood sampling*

Participants were directly posted a commercially available home blood testing kit (Thriva, UK) to measure triglyceride and cholesterol concentrations, several markers of liver function (gamma-GT, alanine transferase, alkaline phosphatase, globulin, albumin, bilirubin) and HbA1c. Participants were asked to collect the blood sample in the morning following an overnight fast. Briefly, participants used an alcohol wipe to clean one finger, pierced the skin using a lancet, and then filled two 1.5 ml Eppendorf tubes with blood. These tubes were then packaged as instructed and posted the same day to an accredited Thriva laboratory and results were uploaded to an online platform visible to the participant and the lead researcher.
