# Supplementary material for: A free-living, walking-based, exercise programme, with exercise timed relative to breakfast, to improve metabolic health in people living with overweight and obesity: A feasibility study
Source: PLoS One. 2024 Nov 21;19(11):e0307582. doi: 10.1371/journal.pone.0307582 (PMC11581328; doi:10.1371/journal.pone.0307582)
Supplement: S2 File — (DOCX) [file pone.0307582.s002.docx]

***Supplementary Table 1.*** Reporting the total duration and mean HR for every 2-week period of continuous session.

| **Variable** | **Continuous** | | | | | | |
| --- | --- | --- | --- | --- | --- | --- | --- |
| Weeks | | 1 + 2 | 3 + 4 | 5 + 6 | 7 +8 | 9 + 10 | 11 + 12 |
| **Duration (min:sec)** | |  |  |  |  |  |  |
| FAST | | 31:27 ± 2:26 | 35:54 ± 2:48 | 42:05 ± 2:51 | 45:45 ± 2:44 | 56:34 ± 11:00 | 01:01:39 ± 4:02 |
| FED | | 34:08 ± 8:56 | 39:04 ± 10:06 | 43:00 ± 5:40 | 30:08 ± 4:57 | 55:33 ± 9:39 | 01:02:11 ± 5:15 |
| **HR_mean_ (% HR_max_)** | |  |  |  |  |  |  |
| FAST | | 69 ± 7 | 69 ± 8 | 69 ± 7 | 69 ± 7 | 69 ± 7 | 68 ± 6 |
| FED | | 70 ± 5 | 69 ± 6 | 69 ± 7 | 68 ± 5 | 67 ± 6 | 67 ± 6 |

***Supplementary Table 2.*** Reporting the total duration, peak HR and time above HR max for every 2-week period of interval session.

| **Variable** | **Interval** | | | | | | |
| --- | --- | --- | --- | --- | --- | --- | --- |
| Weeks | | 1 + 2 | 3 + 4 | 5 + 6 | 7 + 8 | 9 + 10 | 11 + 12 |
| **Duration (min:sec)** | |  |  |  |  |  |  |
| Fasted | | 32:59 ± 4:34 | 36:35 ± 01:54 | 44:06 ± 3:28 | 49:44 ± 6:21 | 55:11 ± 4:08 | 1:05:36 ± 13:05 |
| Fed | | 33:40 ± 8:47 | 39:37 ± 12:04 | 44:48 ± 7:23 | 50:33 ± 5:41 | 55:20 ± 7:35 | 1:02:11 ± 5:15 |
| **HR_peak_ (% HR_max_)** | |  |  |  |  |  |  |
| Fasted | | 81 ± 8 | 82 ± 10 | 82 ± 9 | 83 ± 8 | 84 ± 9 | 83 ± 8 |
| Fed | | 82 ± 4 | 88 ± 24 | 85 ± 9 | 82 ± 6 | 81 ± 9 | 81 ± 8 |
| **Time above 80% HR_max_** | |  |  |  |  |  |  |
| Fasted | | 1:11 ± 2:24 | 1:29 ± 3:40 | 1:31 ± 3:11 | 2:04 ± 4:13 | 1:45 ± 3:12 | 1:55 ± 3:36 |
| Fed | | 0:43 ± 1:09 | 1:29 ± 1:40 | 2:24 ± 1:40 | 1:27 ± 2:16 | 1:14 ± 1:59 | 1:36 ± 2:14 |

Data are mean ± SD of time spent for each variable.

***Supplementary Table 3.*** Energy and macronutrient intake at breakfast, lunch and dinner consumed over the 3 recorded days at baseline and follow-up.

| Baseline – Follow-up | FAST | | FED | |
| --- | --- | --- | --- | --- |
| (g, unless stated) | Baseline | Follow-up | Baseline | Follow-up |
| ***Breakfast*** |  |  |  |  |
| Carbohydrate | 45 ± 22 | 43 ± 20 | 39 ± 20 | 45 ± 39 |
| Fat | 13 ± 11 | 13 ± 10 | 11 ± 9 | 11 ± 8 |
| Protein | 16 ± 11 | 17± 11 | 14 ± 10 | 17 ± 11 |
| Total Energy (kcal) | 355 ± 193 | 347 ± 176 | 309 ± 135 | 324 ± 141 |
| ***Lunch*** |  |  |  |  |
| Carbohydrate | 78 ± 42 | 74 ± 36 | 74 ± 38 | 76 ± 45 |
| Fat | 22 ± 12 | 19 ± 9 | 20 ± 12 | 21 ± 14 |
| Protein | 26 ± 11 | 23 ± 9 | 24 ± 11 | 24 ± 12 |
| Total Energy (kcal) | 607 ± 284 | 562 ± 251 | 567 ± 265 | 586 ± 316 |
| ***Dinner*** |  |  |  |  |
| Carbohydrates | 98 ± 31 | 102 ± 28 | 106 ± 43 | 107 ± 42 |
| Fat | 35 ± 12 | 38 ± 12 | 37 ± 13 | 38 ± 14 |
| Protein | 41 ± 15 | 38 ± 12 | 44 ± 16 | 45 ± 16 |
| Total Energy (kcal) | 866 ± 183 | 861 ± 165 | 926 ± 237 | 995 ± 374 |
| ***Total*** |  |  |  |  |
| Carbohydrates | 221 ± 95 | 219 ± 84 | 219 ± 101 | 228 ± 126 |
| Fat | 70 ± 35 | 70 ± 31 | 68 ± 34 | 70 ± 36 |
| Protein | 83 ± 37 | 78 ± 32 | 82 ± 37 | 86 ± 39 |
| Total Energy (kcal) | 1828 ± 660 | 1770 ± 592 | 1802 ± 637 | 1905 ± 831 |
|  |  |  |  |  |

Data are mean ± SD for mean macronutrient intake in grams.
